# Influence of Body Position on Severity of Obstructive Sleep Apnea: A Systematic Review

**DOI:** 10.1155/2013/670381

**Published:** 2013-10-08

**Authors:** Akshay Menon, Manoj Kumar

**Affiliations:** Cortland ENT and Sleep Lab, Cortland, NY 13045, USA

## Abstract

*Aim.* The aim of this review is to determine the relationship between sleeping body posture and severity of obstructive sleep apnea. This relationship has been investigated in the past. However, the conclusions derived from some of these studies are conflicting with each other. This paper intends to summarize the reported relationships between sleep posture and various sleep indices in patients diagnosed with sleep apnea. *Methods and Materials.* A systematic review of the published English literature during a 25-year period from 1983 to 2008 was performed. *Results.* Published data concerning the sleep apnea severity and posture in adults are limited. Supine sleep posture is consistently associated with more severe obstructive sleep apnea indices in adults. However, relationship between sleep apnea severity indices and prone posture is inconsistent.

## 1. Background

A few prior studies have addressed the relationship between body posture and sleep apnea. It has been observed that the avoidance of supine position leads to a decrease in the number and severity of obstructive episodes [1, 2]. In supine posture, the upper airway caliber and resistance are greater [3, 4]. Also, the tendency for the upper airway to collapse further is greater in supine position compared to lateral position [5, 6]. In patients with mild obstructive sleep apnea, symptomatic improvement may be achieved, simply by avoiding supine posture during sleep. In some patients, avoiding supine position while sleeping may be the only treatment required [7]. 

Some previously published studies have classified OSA patients into two groups: *positional* and *nonpositional* [8]. Positional patients are those in whom the respiratory disturbance index was more than twice as high in the supine position as it was in the lateral position. Nonpositional patients are those in whom supine RDI was less than two times higher than the lateral RDI. The prevalence of positional patients among those with OSA varies from 9% to 60% [9–12]. In general, positional patients are thinner and younger and have less severe breathing abnormality indices compared to nonpositional patients. Also, patients with positional sleep apnea have smaller neck circumference and spend more time in the supine posture as a percentage of total sleep time [13]. Positional sleep apnea was reported as significantly more common when sleep apnea was mild than when it was moderate or severe [12, 13]. The optimal CPAP pressures required for positional patients were significantly lower than that for nonpositional patients [14].

The effect of prone sleep posture on the frequency and severity of apneic episodes has not been as well studied as other postures. We questioned whether respiratory indices during lateral versus prone posture sleep may differ. Most of the time, during sleep in a prone position, the neck is rotated and the head is positioned laterally. When a person sleeps on his/her side, both the body and head are positioned laterally. Turning from the prone posture to the side or supine position decreases the upper airway size in nonpositional patients by decreasing the lateral distance, while the opposite effect was found in the positional patients [15]. Further understanding of postural influence on the severity of sleep apnea may help in identifying therapeutically favorable interventions. The objective of the study is to summarize the established relationships between sleep posture and severity of obstructive sleep apnea and to identify associations that require further elucidation.

## 2. Mechanisms

The anatomical and physiological mechanisms responsible for the improvement in breathing indices while changing from nonsupine to supine posture have neither been clearly described nor completely understood. The following observations were made by various authors in an attempt to elucidate the posture dependent changes to upper airway in patients with obstructive sleep apnea. By using acoustic reflection technique, a decrease in pharyngeal area was seen when patients with OSA changed their body posture from sitting to supine [16]. A cephalometric study of patients with OSA showed an increase in uvular width and narrowing of retroglossal airway by adopting a supine posture [17]. In comparison with normal subjects, OSA patients had significantly longer and wider uvulas with a tendency to have a shorter distance between the uvular protrusion and pharyngeal wall [18]. However, all these studies were done in awake patients.

Some previous studies have also described the differences in anatomical factors between the positional and nonpositional patients. Positional patients have larger posterior airway space, less elongated soft palate, and more prominent retrognathia than nonpositional patients. The minimal cross-sectional area of the upper airway (retropalatal area) of the positional patients was almost twice that of nonpositional patients in both the supine and right lateral position [15]. The shape of the upper airway was more elliptical in positional group compared to circular in nonpositional group. The difference in shape was predominantly due to a greater lateral diameter in positional patients. This will allow them to sleep in the lateral position without significant collapse of the airway. In contrast, other authors have observed an elliptical pharynx in patients with OSA, oriented in the anteroposterior dimension, due to collapsible lateral wall [19]. Another factor, lung volume, decreases during sleep [20], especially in the supine posture which in turn will have a secondary effect of increasing upper airway resistance.

## 3. Material and Methods

We reviewed the existing literature pertaining to body posture that has a significant influence on the severity of obstructive sleep apnea with emphasis on prone posture. The comprehensive search of the published literature was carried out using data stored in “PubMed,” “Embase,” and Cochrane data bases as of July 2008, for a period of 25 years from 1983 to 2008. Search terms used included medical subject headings (MESH) and keywords. The following key words are used alone and in combination: sleep, apnea, posture, position, supine, lateral, prone, positional, and nonpositional. Additionally, these databases were searched with the names of researchers in the field to see whether there were additional relevant papers from these authors. Article titles, abstracts, and reference sections were initially reviewed to determine article eligibility and also to identify other relevant studies. Non-English language was not used as exclusion criteria; however, no eligible studies in other languages were identified. 

A Google Scholar advanced title search was also carried out for a period between 1983 and 2008. The search was limited to articles published under the subject headings of “medicine and pharmacology.” The words “posture” and “obstructive sleep apnea” were used in combination to perform the search. The titles of the cited articles were also reviewed manually using the links given in Google Scholar.

We used the following criteria to include a study in our review.

1. The study must be published as a journal article between 1983 and 2008.
2. Subjects of the study must be evaluated with nocturnal polysomnogram to include at a minimum the measurement of apnea or apnea hypopnea index.
3. All studies which have documented apnea hypopnea index for at least two different postures were included. Apnea was defined as an episode of complete cessation of breathing for at least 10 seconds. Hypopneas were defined as a reduction in airflow accompanied by a desaturation of 3% or more and/or arousal.
4. Apnea hypopnea index or apnea index should be more than 5 for adult patients and more than 1 for pediatric patients.

## 4. Results 

13 studies were selected after an initial review of title and abstract (Table 1) which appeared to fulfill the inclusion criteria. All the studies had some shortcomings, such as small sample size, underreporting of methods and data, and lack of randomization and blinding. However, in view of the limited number of articles found, it has been decided to review all these manuscripts in detail. Search of the Cochrane database did not reveal any eligible results.

*Paper No. 1 (Richard et al. [12])*. The paper studied 120 patients with a mean age of 50.1 years. Patients with an AHI of more than 10 or with an AHI between 5 and 10 with an ESS of at least 7 were included in the study. The authors classified the study group into positional and nonpositional patients. 55.8% of patients were position dependent as described above. In the position dependent group, AHI in the supine position was 43 ± 19.4, and in nonsupine position, AHI was 8.2 ± 7.9. In contrast, for the nonpositional patients, the supine AHI was 23.1 ± 23.2 and the nonsupine AHI was 23.9 ± 15.3. The percentage of REM sleep was equal for both groups. The mean REM-AHI index was 25.7, and the mean NREM-AHI index was 21.2.

This paper failed to mention the total mean AHI for all patients together. However, when such a calculation is made from the separate data for positional and nonpositional patients, the following results were obtained. The mean supine AHI for both groups together was 34.2 compared to mean nonsupine AHI of 15.1. These findings confirm the advantage of nonsupine sleep position for patients with obstructive sleep apnea.

*Paper No. 2 (Pereira et al. [21])*. This study involved 60 patients younger than 3 years, with a mean RDI score of 10.8 ± 3.4. For the supine sleep, the mean RDI was 18.5 ± 5.1 compared to 7.2 ± 1.9 for the nonsupine sleep. The REM-RDI was 20.5 compared to 5.6 of NREM-RDI. The paper did not analyze the prone posture separately. The supine sleep was recorded on average of 40% of the time. This study also confirms the advantage of nonsupine sleep.

*Paper No. 3 (Mador et al. [13])*. This two-center study involved a total of 269 patients, of which only 117 patients had a positive sleep study. Study included patients who slept for at least 15 minutes each in supine and nonsupine position. Only 14 patients had sufficient sleep in both postures during REM sleep. The nonsupine AHI for REM sleep was 10.3 ± 9.5 (center 1) and 3.4 ± 3.6 (center 2). The supine AHI for REM sleep was 33.9 ± 27.1 (center 1) and 45.7 ± 28.1 (center 2). The patients with nonpositional sleep apnea had a significantly greater AHI. AHI was also greater in supine posture than nonsupine.

*Paper No. 4 (Cuhadaroglu et al. [22])*. 55 children with a mean age of 5.5 ± 1.5 were included. Patients with a body mass index higher than the 85th percentile were excluded, reducing the study group size to 33. Children were again subclassified into those with and without adenoid and tonsillar hypertrophy for data analysis. However considering all 32 children together, the mean AHI for supine, lateral, and prone was 9.28, 14.6, and 6.05, respectively. In children with adenoid and tonsillar hypertrophy, supine posture was the worst, but for the rest, it was the lateral position. For all groups, sleeping in prone position showed some advantage over supine and lateral positions.

*Paper No. 5 (Akita et al. [23])*. 372 subjects were involved with a mean age of 47 years. The AHI was significantly better in lateral posture, compared to supine. This association was stronger in patients with less severe OSA and/or lower BMI. No posture dependent specific data is available from the study. 

*Paper No. 6 (do Prado et al. [24])*. Sleep studies from 80 children with a mean age of 4.9 years and an AHI of ≥1 were analyzed. The relative risk of sleep apnea in the prone position was 1.68 compared to the supine position. The mean AHI for the supine, lateral, and prone postures was 7, 8, and 12, respectively. For the REM sleep, these figures were 24, 23, and 21, respectively. The mean apnea duration was 12 seconds in prone position and 11 seconds in the lateral and supine positions. The mean time for the study group spent in prone posture was only 10 minutes compared to 252 minutes in the lateral and 81 minutes in the supine postures. 

*Paper No. 7 (Matsuzawa et al. [25])*. This is a case report of a 45-year-old man with history suggestive of sleep apnea. The patient slept mostly in the lateral and in the supine positions (76.3% and 21.3%, resp.), although for a period of time, he spent 11 minutes (2.3%) contiguously in the prone position. The mean AHI for supine, lateral, and prone postures was 78, 63.4, and 0, respectively. 

*Paper No. 8 (Cartwright et al. [26])*. 60 patients with a mean age of 48.9 were included, but only 24 patients slept on both back and side in both REM and NREM sleep stages. The analysis of these 24 patients revealed that AHI is twice as high in the supine compared to the lateral sleep posture. There is also a significant increase in the frequency of apneic events in the REM sleep. 

*Paper No. 9 (Miki et al. [27])*. 7 male patients were included. The mean apnea index (AI) for the supine posture was 51 ± 8.6. The mean for the lateral position was 27.6 ± 9.6. 

*Paper No. 10 (George et al. [10])*. Seven patients were studied. AHI was greater on the back than on the sides (84.4 ± 4.9 versus 73.6 ± 7.5), but after accounting for sleep stage, this difference remained only for NREM stage sleep and not for REM. 

*Paper No. 11 (Chaudhary et al. [28])*. This is a two-stage study of 4 patients. A PSG in the supine posture was initially obtained followed by one in the lateral position. The mean AI was 33 in the first sleep study which has dramatically reduced to 5 in the lateral position. 

*Paper No. 12 (Kavey et al. [7])*. This is a study of 4 patients with multiple polysomnograms. Only two patients of a total of four spent some sleep time in prone, and only one patient slept in all the three positions during one of the 3 sleep studies he had undergone. The AI was 22.5 for the supine position and 0 for both lateral and prone positions. That patient, however, slept on both lateral and prone postures during the other two nights, with a mean AI of 22.5 (prone) and 0.65 (lateral). The PSG of the second patient who slept in both prone and lateral positions showed an AI of 14.3 (prone) and 0.6 (lateral). 

*Paper No. 13 (Cartwright et al. [1])*. 30 patients were studied, but only 24 slept in more than one posture. Only 3 patients spent any time in the prone posture, and prone data were not reported. The mean total AHI was 47.5 ± 35.4. The supine AHI was 63.5 ± 39 and the lateral was 37.8 ± 37.5. 

## 5. Discussion

Summarizing the published literature to determine the influence of prone posture in sleep apnea is difficult given the dearth of definitive studies. Among the thirteen studies examined, only three evaluated prone posture. The data for two of these studies were collected from pediatric age groups, and the third study was a description of a single adult patient in the form of a case report. The main finding of do Prado et al. [24] was that children with OSA breathed best when supine. In children with OSA, the airway obstruction usually occurs at the levels of adenoid or soft palate rather than at the level of base of the tongue [29]. Gravity is less important in children with adenoid hypertrophy, as adenoids are more fixed than tonsils. Therefore, it is speculated that children with adenoid hypertrophy and OSA may do better in supine than in lateral posture [22]. Prolapse of the tongue in supine position is therefore not as important as in adults. In children, the lung capacity may become reduced in prone position due to pressure on a compliant thorax which in turn may lead to narrowing of the upper airway [30]. 

In contrast, Cuhadaroglu et al. [22] demonstrated significant improvement in AHI in prone position. However, they did not report the position of the neck and therefore were unable to provide an explanation for the improvement observed in prone posture. Their data also showed that the mean AHI in the lateral position was higher than that observed in the supine. In children under general anesthesia, prone position decreases the maximal distension of the most collapsible region of the pharynx, and the associated neck rotation increases pharyngeal closing pressure compared with supine position [31]. 

In an MRI scan study of pediatric population below 10 years, the mean total airway volume was 6.0 ± 2.9 mL in the supine position compared to 8.7 ± 2.5 mL in the lateral position [32]. Also, all noncartilaginous segments of the upper airway increased in area in the lateral compared with the supine position. 

In a recent study of children younger than 3 years, the supine RDI (18.5 ± 5.1) was found to be significantly greater than the total RDI (7.2 ± 1.9) [21]. The authors suggest that toddlers may have sleep characteristics that are different from those of older children. In contrast, there are other studies which have failed to support such a correlation [24]. Thach and Stark suggested that infants sleeping in prone or lateral position flex their neck, which in turn can lead to an increased upper airway collapsibility [33]. Interestingly, there are studies which demonstrate an increase in upper airway collapsibility in prone position in infants [31]. 

Eleven of the thirteen papers reviewed revealed that the numbers of obstructive respiratory events during sleep were reported to be fewer with the lateral or nonsupine position than the supine position in patients with OSA. The improvement achieved in lateral position may be due to a combination of mechanisms including structural alteration of the airway in the lateral position and increased activity of pharyngeal dilator muscles. 

Isono et al. [29] found that the position change from the supine to the lateral enlarged both retropalatal and retroglossal airways and decreased closing pressure at both sites by approximately 3 cm H_2_O on average in completely paralyzed and anesthetized patients with OSA [34]. However, a number of other studies [35, 36] demonstrated a similar reduction of closing pressure in the lateral position regardless of the condition of the upper airway muscle activities, indicating that the neural mechanisms may contribute little to the improvement of pharyngeal airway by the lateral position. Oksenberg et al. [11] demonstrated that the apneic events occurring in the supine posture are more severe than the apneic events occurring in the lateral posture [8]. The authors evaluated apnea duration, nadir desaturation, duration of arousal, and maximum snoring loudness in both postures and found them to be of greater severity in supine position than those occurring in lateral position. These findings were consistent with both positional and nonpositional patients. These observations are in agreement with a number of other published papers [37].

Matsuzawa et al. [25] reported a patient with OSA whose apnea hypopnea index (AHI) improved remarkably in the prone position accompanied by an improved sleep quality [25]. The author performed an MRI scan of the upper airway while the patient was awake to determine the posture associated changes in the upper airway. Oropharyngeal stenosis was most severe in the supine posture due to posterior prolapse of the tongue and soft palate. It is likely that the gravity allows the tongue to fall backwards against the pharyngeal wall. The predictability of response to a tongue retaining device (TRD) depends on the degree of collapse of the tongue and the ability of a TRD to prevent such a prolapse [38].

Tsuiki et al. [39] demonstrated that the velopharynx is not only the narrowest site in both upright and supine body positions but also the most changeable site in response to an alteration in body position in awake OSA patients. The authors performed upright and supine cephalometries to derive this conclusion [39]. Similar findings were recorded by other authors as well [40]. 

Positional training to avoid supine posture can improve subjective sleepiness, maintenance of wakefulness time, and psychometric test performance to a similar degree as CPAP, but the latter was more effective in reducing apnoea/hypopnoea and oxygen desaturations [41]. Various estimates indicate that positional therapy alone could be used to treat approximately 30 to 50% of all patients with OSA [9]. Analysis of data from CPAP titration study has shown that the mean optimal CPAP pressure was significantly higher in the supine posture than in the lateral posture [42].

Effect of posture on REM sleep is different from that of on non-REM sleep [15]. It was reported that the increase in AHI in supine position compared to lateral position was evident only in NREM sleep [10]. Cartwright et al. showed that patients with positional sleep apnea prefer the lateral position over the supine position in REM sleep [26]. Duration of apnea was longer in REM sleep regardless of the body position [10].

A reduction in the prevalence of *positional patients* was noted with an increase in BMI [42]. The same authors also found that *nonpositional* obese patients became *positional* on losing weight. The improvement due to changes in posture became increasingly insignificant with increase in BMI [23]. The prevalence of positional patients was found to be less in older patients with OSA compared to their younger counterparts [42]. 

Accuracy in recording of sleep posture is a variable to be considered. Direct review of videotape recordings of the patient appears to yield more consistent records of sleep posture than that of various digital position monitors. Real-time posture scoring by the nighttime technicians also appeared to be highly accurate and is an acceptable method for posture scoring [13]. When split-night studies were performed, sufficient sleep in both postures was rarely observed, making it impossible to assess for a positional component in most cases [13]. 

In a study to determine if physical constraint due to the polysomnography apparatus affects sleep study results by inducing subjects to sleep in the supine position, the authors observed that the time spent in supine position was 56% greater during sleep study nights with attached leads [43] than when no PSG leads are attached. In addition, there are some data suggesting that patients spend more time in the supine posture during polysomnography compared to home sleep [18]. Hence, the severity of OSA may be overestimated in the laboratory settings. The sleep study centers need to recognize this possibility and should advise their patients to occupy their natural sleep postures during the study. 

## 6. Summary 

Review of the published literature to determine the association between posture and OSA identified only thirteen articles with relatively small sample sizes. A critical reader may be dismayed by this fact; nevertheless, the findings are noted to be consistent in all of the reviewed studies except two. Supine sleep posture is consistently associated with more severe obstructive sleep apnea indices in adults, but this appears to be less consistent in pediatric patients. Published data concerning adults in prone position are limited and not consistent [7]. Further study of prone position in adults is needed.

## Figures and Tables

**Table 1:** The brief summary of the articles reviewed.

Author	Year	Sample size	Mean age	Mean RDI supine	Mean RDI prone	Mean RDI lateral	Findings
Richard	2006	120	50	34		15	Supine worse than nonsup
Pereira	2005	60	3	18		7	Supine worse than nonsup
Mador (REM)	2005	269	55	39		7	Supine worse than nonsup
Cuhadaroglu	2003	55	5	9	6	15	Prone better than nonprone
Akita	2003	372	47				Lateral better than supine
Fernandes do Prado	2002	80	5	7	12	8	Prone worse than nonprone
Matsuzawa	1995	1	45	78	0	63	Prone better than nonprone
Cartwright (NREM)	1991	60	50	61		12	Supine worse than nonsup
(REM)		60	50	80		32	Supine worse than nonsup
Miki	1988	7		51		28	Supine worse than nonsup
George	1988	7	45	84		74	Supine worse than nonsup
Chaudhary	1986	4	47	33		5	Supine worse than nonsup
Kavey	1985	4	52	∗			Supine worse than nonsup
Cartwright	1984	30		64		38	Supine worse than lateral

Mean age and RDI are rounded to nearest whole number. *Kavey et al. had data from two patients who slept in prone posture which is described in the results section.
